# Gray Matter Features of Reading Disability: A Combined Meta-Analytic and Direct Analysis Approach^1^,^2^,^3^,^4^

**DOI:** 10.1523/ENEURO.0103-15.2015

**Published:** 2016-01-23

**Authors:** Mark A. Eckert, Virginia W. Berninger, Kenneth I. Vaden, Mulugeta Gebregziabher, Loretta Tsu

**Affiliations:** 1 Department of Otolaryngology, Head and Neck Surgery, Medical University of South Carolina, Charleston, South Carolina 29425; 2 Department of Education, University of Washington, Seattle, Washington 98195; 3 Department of Public Health Sciences, Medical University of South Carolina, Charleston, South Carolina 29425

**Keywords:** dyslexia, multisite, orbitofrontal gyrus, reading disability, superior temporal sulcus, voxel-based gray matter

## Abstract

Meta-analysis of voxel-based morphometry dyslexia studies and direct analysis of 293 reading disability and control cases from six different research sites were performed to characterize defining gray matter features of reading disability. These analyses demonstrated consistently lower gray matter volume in left posterior superior temporal sulcus/middle temporal gyrus regions and left orbitofrontal gyrus/pars orbitalis regions. Gray matter volume within both of these regions significantly predicted individual variation in reading comprehension after correcting for multiple comparisons. These regional gray matter differences were observed across published studies and in the multisite dataset after controlling for potential age and gender effects, and despite increased anatomical variance in the reading disability group, but were not significant after controlling for total gray matter volume. Thus, the orbitofrontal and posterior superior temporal sulcus gray matter findings are relatively reliable effects that appear to be dependent on cases with low total gray matter volume. The results are considered in the context of genetics studies linking orbitofrontal and superior temporal sulcus regions to alleles that confer risk for reading disability.

## Significance Statement

Developmental reading disability limits educational, social, and professional achievement for ∼5–15% of the U.S. population. The neuroanatomical bases for reading disability have been unclear, in part because of the heterogeneous nature of this complex disorder. A combined meta-analysis and direct data analysis of a large multisite dataset revealed consistent left orbitofrontal and left superior temporal sulcus gray matter volume effects in reading disability compared to control cases that rise above the considerable behavioral and anatomical variance in reading disability samples. Importantly, these gray matter predictors of reading disability have been significantly associated with genetic markers that confer risk for reading disability. Thus, the results demonstrate brain regions that are consistently affected in the heterogeneous reading disability population.

## Introduction

Reading disability affects at least 5–15% of the U.S. population (Shaywitz et al., 1990; Katusic et al., 2001; Yoshimasu et al., 2010) for which there are varied patterns of oral and written language problems (Torppa et al., 2007; Archibald et al., 2013; McArthur et al., 2013). There has been progress toward understanding the neuroanatomical bases for reading disability (Vandermosten et al., 2012; Richlan et al. 2013), but small sample sizes and uncontrolled demographic factors limit interpretation of reading disability findings. Meta-analysis and direct analysis of large multisite datasets can help to move beyond these limitations to advance our understanding of reading disability.

Meta-analyses of findings from voxel-based gray matter studies of reading disability (Linkersdörfer et al., 2012; Richlan et al., 2013) suggest that people with reading disability exhibit lower gray matter volume at the posterior end of the Sylvian fissure, the fusiform gyrus, and cerebellar regions where gross volumetric and/or atypical asymmetry effects have also been observed (Leonard et al., 2001; Rae et al., 2002; Fernandez et al., 2013). Each of these brain regions has been linked to reading and/or reading disability in functional imaging studies (Maisog et al., 2008; Paulesu et al., 2014) and are targets for understanding reading disability.

One limitation of the meta-analysis approach is that group effects can be dependent on particular characteristics of the samples or experimental design of each study. This is important because voxel-based gray matter findings in dyslexia studies appear to be affected by demographic variables, such as gender (Altarelli et al., 2013, 2014; Evans et al., 2014). In addition, gray matter measures normally covary with gender and age (Barnea-Goraly et al., 2005; Wilke et al., 2007; Witte et al., 2010). These demographic influences on brain morphology may contribute to inconsistent results across studies with varying age and gender distributions (Eckert et al., 2005; Tamboer et al., 2015), particularly when small sample sizes are studied (*N* = 8–46, median = 13; Brown et al., 2001; Brambati et al., 2004; Eckert et al., 2005; Silani et al., 2005; Vinckenbosch et al., 2005; Hoeft et al., 2007; Kronbichler et al., 2008; Menghini et al., 2008; Steinbrink et al., 2008; Raschle et al., 2011; Jednoróg et al., 2013; Krafnick et al., 2014; Tamboer et al., 2015) for a disorder that is anatomically heterogeneous (Leonard et al., 2001).

Integrating existing data across research sites can address the challenges noted above by increasing statistical power, but can be limited when investigators use different recruitment strategies. Children with dyslexia are typically included in studies if they exhibit impaired phonological processing based on pseudoword decoding and real word reading measures (Fletcher, 2009). Impairments in these core reading skills are also likely in children with specific language impairment or oral and written language learning disability (OWL LD) who exhibit problems in listening comprehension, reading comprehension, oral expression, and/or written expression (Silliman and Berninger, 2011). Distinguishing behavioral features of OWL LD are not always measured (Pennington and Bishop, 2009) and this increases the likelihood that reading disability samples include children with OWL LD and dyslexia, especially when participants are recruited from clinical settings (Catts et al., 2005).

Children with OWL LD often have below average verbal comprehension, (Silliman and Berninger, 2011) and low brain volume (specific language impairment; Leonard et al., 2006; Girbau-Massana et al., 2014). Low brain volume in children with low verbal comprehension appears be expressed before children learn to read and differentiates children with and without receptive language impairment within a reading disability sample (Kibby et al., 2009). In addition, there is evidence of below average head circumference at birth in children with specific language impairment compared to controls (Whitehouse et al., 2012) and a positive association between infant variation in brain size and childhood intelligence (Gale et al., 2006). Together, these findings suggest that verbal comprehension and brain volume can be used to characterize evidence for OWL LD in reading disability samples.

The current multisite study included data from research sites where verbal comprehension was measured so that the impact of verbal comprehension and brain volume on reading disability group differences could be examined. We addressed this and the other methodological concerns noted above to test the hypothesis that brain regions supporting reading are atypical in children with reading disability (Richlan, 2012). An integrated meta-analytic and direct voxel-based analysis of multisite data provided increased statistical power with the goal of establishing reliable predictors of reading disability that are targets for understanding the etiologies and treatment of reading disability.

## Materials and Methods

### Voxel-based meta-analysis

A Signed Differential Mapping (SDM v4.13; Radua et al., 2012) meta-analysis was performed to identify consistent voxel-based gray matter effects across reading disability studies (Table 1). This analysis included 58 peak coordinates that were weighted by the statistical thresholds used to identify the reported effects from comparisons of 218 control and 244 dyslexia cases across eleven publications (Brown et al., 2001; Brambati et al., 2004; Eckert et al., 2005; Silani et al., 2005; Vinckenbosch et al., 2005; Kronbichler et al., 2008; Menghini et al., 2008; Steinbrink et al., 2008; Raschle et al., 2011; Jednoróg et al., 2013; Tamboer et al., 2015). These papers were identified based on PubMed searches for dyslexia and reading disability voxel-based gray matter studies published through the end of April 2015 and included a study of 5- to 6-year-old children who were and were not at risk for dyslexia (Raschle et al., 2011). Studies involving cases with impaired learning of a logographic written language system were excluded. Studies by Hoeft et al. (2007) and Krafnick et al. (2014) were also excluded from the meta-analysis because the principal investigator from these studies contributed data to this project. Excluding these two studies from the meta-analysis ensured that the meta- and direct data analyses described below were performed with completely independent datasets.

**Table 1.** Summary information for manuscripts included in the meta-analysis

First author	Control sample size	Dyslexic sample size	Coordinate space	Mean age dyslexia cases	Gender ratio M/F	Statistical significance
Brambati et al., 2004	11	10	Talairach	31.6	47	0.006
Brown et al., 2001	14	16	Talairach	24.5	30	0.05
Eckert et al., 2005	13	13	MNI	11.4	100	0.001
Jednoróg et al., 2013	35	46	MNI	10.3	48	0.00004
Kronbichler et al., 2008	15	13	MNI	15.9	100	0.004
Menghini et al., 2008	10	10	MNI	40.7	10	^0.005
Raschle et al., 2011	10	10	MNI	5.9	50	0.0002
Silani et al., 2005	32	32	Talairach	∼24.4	100	0.009
Steinbrink et al., 2008	8	8	MNI	20.1	75	^0.05 FDR
Tamboer et al., 2015	37	57	MNI	20.6	7	^0.05
Vinckenbosch et al., 2005	14	10	Talairach	17–30	100	0.0006

Thresholds are reported as uncorrected thresholds based on the reported t or Z-scores, except for ^ three studies for which only the statistical threshold was reported. Ages were rounded or ∼ estimated from the mean age of multiple groups or reported as a range if mean ages were not provided.

SDM was used because the method incorporates the effect sizes from studies in the meta-analysis. The statistical thresholds, determined based on the reported statistics in each study, ranged from *p* < 0.05 to *p* < 0.00004 (uncorrected) for the result with the smallest effect size reported in each study. Conversion between reported *t* scores, *p* values, and *Z*-scores was performed using the SDM “Statistics converter” functions (http://www.sdmproject.com/utilities) for all studies except one (Steinbrink et al., 2008) that used a false discovery rate (FDR)-corrected threshold and did not report group difference statistics. We assumed a large effect size for this study (*p* < 0.0001) given the relatively small sample size (*n* = 16) and significant effects for the FDR *p* < 0.05 threshold.

The meta-analysis included 500 permutations and a default 20 mm isotropic kernel parameter. These methods are consistent with those used by Richlan et al. (2013), who used a *p* < 0.005 uncorrected statistical threshold for defining consistently significant effects across studies. The results below are presented with *p* < 0.005 and *p* < 0.001 uncorrected thresholds, with the *p* < 0.001 threshold used to define regions-of-interest (ROIs) for the direct analysis of the multisite sample data.

### Multisite samples for gray matter analyses

Data from six neuroimaging studies designed to examine reading disability and control group differences in brain structure and/or function were collected as part of a larger study on developing methods for multisite studies. Identification of the reading disability cases across contributing sites included recruitment from clinical settings, schools specializing in dyslexia remediation, and the local community. Institutional Review Board approval was obtained to receive each de-identified dataset at the Medical University of South Carolina and approval for sharing de-identified data were obtained at the institution of each contributing site. The research presented here was performed in accordance with the provisions of the Declaration of Helsinki. Summary statistics for demographic and reading performance information within and across research sites are presented in Table 2.

**Table 2.** Control and reading disability behavioral summaries (mean and SD) before (*n*=293) and after (*n*=255) discriminant function selection of cases

Site	Control					Reading disability				
(*n*=Control/RD) (*n*=129/164)	VC	WID	WA	PC	Age	VC	WID	WA	PC	Age
1 (*n*=36/45)	107.81 (14.19)	105.56 (11.89)	105.61 (10.26)	101.74 (10.26)	10.11 (1.38)	91.40 (14.33)	82.31 (10.22)	87.18 (8.41)	84.73 (12.35)	9.91 (1.65)
2 (*n*=34/28)†	105.86 (4.03)	110.85 (10.18)	110.24 (11.37)	112.94 (8.65)	12.51 (3.06)	106.01 (4.04)	81.93 (7.58)	87.79 (7.58)	87.11 (11.24)	14.27 (1.68)
3 (*n*=15/15)^	117.40 (14.97)	117.07 (17.44)	115.60 (15.98)	109.93 (9.39)	9.33 (2.02)	96.75 (8.55)	84.13 (7.51)	86.60 (7.69)	82.53 (9.57)	9.27 (2.02)
4 (*n*=10/16)	121.09 (16.41)	106.80 (17.71)	118.10 (15.24)	111.10 (14.36)	11.93 (1.89)	102.20 (13.73)	82.44 (8.86)	88.13 (8.07)	84.66 (9.47)	9.99 (1.87)
5 (*n*=19/39)	115.84 (14.07)	115.47 (14.16)	112.42 (12.38)	107.63 (13.59)	9.37 (2.66)	107.73 (11.07)	77.44 (9.56)	89.03 (10.11)	76.87 (15.89)	10.10 (1.67)
6 (*n*=15/21)‡	117.66 (12.75)	114.80 (7.51)	112.93 (9.10)	94.06 (4.09)	10.71 (3.42)	106.85 (13.43)	95.19 (11.51)	98.90 (9.57)	93.94 (3.82)	10.82 (3.49)
**All cases**	111.77 (13.39)	110.92 (13.18)	110.82 (12.34)	106.35 (11.76)	10.75 (2.73)	101.30 (13.39)	82.91 (10.69)	89.26 (9.44)	84.24 (12.88)	10.77 (2.59)
**Discriminant function analysis selected groups** (*n*=Control/RD) (*n*=105/150)										
1 (*n*=23/43)	113.30 (11.24)	112.83 (7.89)	111.04 (8.06)	106.38 (9.28)	10.15 (1.38)	89.56 (11.71)	81.58 (9.85)	86.63 (8.19)	83.16 (10.14)	9.90 (1.65)
2 (*n*=32/26)†	105.94 (3.98)	111.94 (9.46)	111.16 (11.06)	113.09 (9.00)	12.52 (3.12)	105.88 (4.03)	80.96 (6.90)	86.96 (7.11)	86.50 (11.40)	14.19 (1.71)
3 (*n*=14/15)^	118.43 (14.97)	118.93 (16.48)	117.64 (14.40)	111.36 (7.89)	9.38 (2.09)	96.75 (8.55)	84.13 (7.51)	86.60 (7.69)	82.53 (9.57)	9.27 (2.02)
4 (*n*=7/16)	123.27 (19.04)	115.57 (10.95)	121.57 (15.14)	117.86 (8.92)	11.54 (2.04)	102.20 (13.73)	82.44 (8.86)	88.13 (8.07)	84.66 (9.47)	9.99 (1.87)
5 (*n*=15/39)	117.13 (14.10)	120.20 (11.94)	116.27 (10.93)	111.63 (12.25)	9.46 (3.00)	107.73 (11.07)	77.44 (9.56)	89.03 (10.11)	76.87 (15.89)	10.10 (1.67)
6 (*n*=14/11)‡	118.51 (12.75)	115.86 (6.54)	113.93 (8.55)	94.10 (4.06)	10.64 (3.54)	107.81 (16.61)	85.91 (6.36)	92.55 (7.50)	94.18 (3.66)	9.98 (3.68)
**Selected cases**	113.65 (12.66)	115.01 (10.74)	113.79 (11.19)	108.97 (10.97)	10.83 (2.90)	100.52 (13.27)	81.06 (9.00)	87.90 (8.49)	83.01 (12.46)	10.65 (2.51)

RD, reading disability; VC, verbal comprehension; WID, word identification; WA, word attack; PC, passage comprehension.

†This site had complete verbal comprehension missingness. Younger controls were recruited to have reading level matches for the reading impaired cases and this produced a group difference in age (*p* < 0.05) that remained significant after discriminant function assignment of cases to reading groups.

^Gender was missing for 1 case.

‡ This site had complete passage comprehension missingness. Significant group differences were observed for all variables between reading disability and control groups (*p* < 0.001), with the exception of age (n.s.). There were no significant differences in the distribution of gender within sites or across the multisite sample before or after discriminant function analysis selection of cases. The standardized scores are age-normed values.

### Multisite behavioral assessment

Children in each sample were administered real word identification (Letter–Word Identification), pseudoword decoding (Word Attack), and reading comprehension (Passage Comprehension cloze task) subtests from the Woodcock–Johnson IIIR (WJIII; 3 sites; Woodcock et al., 2001) and Woodcock Reading Mastery Tests (3 sites; Woodcock, 1987). Verbal comprehension was assessed using the *Wechsler Intelligence Scales for Children* (WISC; 1 site; Wechsler, 2004) and the *Wechsler Abbreviated Scales of Intelligence* (2 sites; Wechsler, 1999). The WISC Vocabulary subtest was administered at five sites. The WISC Similarities subtest was administered at four sites. The WISC Information and Comprehension subtests were administered at two sites. Finally, the Verbal Comprehension score was obtained at one site from an off-site neuropsychologist who referred the children to the study but did not provide subtest scores like Information, Vocabulary, Similarities, and Comprehension that contribute to the overall Verbal Comprehension Factor or Index Score.

There were differences in mean performance between sites for the standardized behavioral scores that appear to reflect site differences (Table 2), rather than test version. For example, word identification performance was significantly different between reading disability groups from two sites where the WJIII was administered (*t*_(63)_ = 4.67, *p* < 0.001). The pattern and severity of impaired reading skills therefore appeared to differ across sites. There was, however, consistently lower real word reading scores than pseudoword reading among reading disability cases across sites (*t*_(160)_ = −10.36, *p* < 0.001). This difference in real word relative to pseudoword reading impairments may indicate the presence of children with OWL LD in the samples (Berninger, 2008; Berninger and May, 2011, their Table 2) and motivated the analyses designed to control for potential OWL LD effects.

### Multisite structural imaging data

Table 3 presents the acquisition parameters that were used to collect T1-weighted images for 306 cases across the six sites. The VBM8 Toolbox image covariance function (http://dbm.neuro.uni-jena.de/vbm/download) and visual inspection were used to identify and exclude 13 cases with poor image quality, resulting in 293 cases for analysis. Each of the images was rigidly transformed into anterior commissure–posterior commissure alignment. The images were then bias field-corrected using SPM8 and denoised using a non-local means filter that estimated and removed Rician noise in the images (Manjón et al., 2010). Probabilities for gray matter, white matter, and CSF in each voxel were estimated using the SPM8 New Segment algorithm. The gray matter probability images were spatially transformed into a study-specific normalized space using a diffeomorphic normalization procedure (SPM, DARTEL default settings; Ashburner, 2007). Each image was modulated to adjust for volumetric change during normalization and smoothed with an 8 mm Gaussian kernel. The normalized, modulated, and smoothed images were used to: (1) obtain average gray matter volume from within the meta-analysis ROI, and (2) perform exploratory voxel-based gray matter analyses. In summary, these methods were chosen to be consistent with commonly used voxel-based morphometry protocols (Kurth et al., 2015).

**Table 3.** T1-weighted image parameters from the six study sites

Site	Manufacturer	Field strength, T	Image dimensions, mm	Slice thickness, mm	TR, ms	TE, ms	Flip angle , deg
1	Siemens	1.5	256 × 256×200	0.80†	25.00	4.60	30‡
2	GE	3.0	256 × 256×124	1.20	9.00	2.00	15
3	Siemens	3.0	240 × 256×176	0.90	2250*	3.96	9
4	Siemens	3.0	128 × 256×256	1.33	6.00	2.90	8
5	Siemens	3.0	256 × 256×160	1.00	1600^	3.37	15
6	Philips	3.0	256 × 256×100	1.00	9.88	4.59	8

†0.8 mm gap.

‡Five of 66 cases had TR= 30 and flip angle=35. Note that the long TRs for Sites 3 and 5 were due to the use of inversion recovery.

*Inversion time = 900.

^Inversion time = 640.

The meta-analysis results were transformed from MNI coordinate space into the study-specific space of the DARTEL template using the default nonlinear normalization procedure in SPM. Specifically, the SPM *a priori* gray matter image was normalized to the DARTEL gray matter template and the normalization parameters were then applied to the SDM meta-analysis results image. MRICRon (http://www.mccauslandcenter.sc.edu/mricro/mricron) was then used to define the space of each SDM cluster or ROI in order to collect each case’s average smoothed gray matter volume from within each ROI using MarsBaR (Brett et al., 2002). These meta-analysis and image processing steps generated 3 gray matter volume ROI variables that were correlated with the 4 behavioral measures and reading groups after controlling for age, age-squared, gender, site, and total gray matter volume. The estimate of total gray matter volume was derived by summing the gray matter probability values from each participant’s native space segmented gray matter image.

Different scanners and acquisition protocols can differentially affect the gray to white matter contrast in T1-weighted images and can therefore affect image processing and voxel-based results. We controlled for differences in T1-weighted contrast across research sites by using research site dummy variables in the analyses described below (Walhovd et al., 2011). These dummy variables together accounted for 95% of the variance for the gray to white matter ratio (GWR) that was collected from the denoised and bias field-corrected T1-weighted images. The GWR measure was obtained by normalizing the original images into the coordinate space of the study-specific template using the DARTEL normalization parameters, using MarsBaR to collect and average the gray or white matter values from across regions that had at least 50% gray or white matter probabilities, and then dividing these two values. We chose to use a dummy variable to represent each site in the statistical analyses described below rather than the GWR measure because the dummy variables could also capture additional potential differences between the sites that were not observable (eg, recruitment strategies).

### Statistical analyses

#### Missingness

Multiple imputation (Rubin, 1996; Parker and Schenker, 2007; Vaden et al., 2012) was used to deal with behavioral data missingness within and across sites (percentage missingness across the multisite sample: Word Attack, 1%; Word Identification, 1%; Passage Comprehension, 14%; Verbal Comprehension, 25%). It is important to deal with missingness rather than use an available case analysis (only cases with data for all variables) because complete case analysis can produce biased results (Rubin, 1996). Specifically, available case analysis produces false negative results (eg, simulation results shown by Vaden et al., 2012, their Fig.5).

Multiple imputation was developed for dealing with missing data that are conditional on the observed data or missing at random (MAR; Little and Rubin, 2002). Although it is difficult to prove MAR, predictors of missingness from the observed data can provide support for the assumption of MAR in the use of multiple imputation with the additional assumption that missingness does not depend on unobserved data. We identified multiple predictors of missingness in our data. For example, children with higher Passage Comprehension scores were more likely to have missing Verbal Comprehension data (*r* = 0.23, *p* = 0.0003) and children with higher Word Identification, Word Attack, and Verbal Comprehension were more likely to have missing Passage Comprehension scores (*r* = 0.17, *p* = 0.004; *r* = 0.14, *p* < 0.043; *r* = 0.14, *p* = 0.043, respectively). Given these associations, we also reasoned that Verbal Comprehension and Passage Comprehension missingness were not because of an intellectual or physical inability to perform the Verbal Comprehension tests [a condition for missing not at random (MNAR)] because the children were able to perform the reading-related tests. Therefore, missingness appeared to be MAR rather than MNAR and appropriately addressed using multiple imputation (Rubin, 1996).

Strong predictors of the observed data were also identified and used to inform the multiple imputation. For example, 78% of the variance in the observed Passage Comprehension and 50% of the variance in the observed Verbal Comprehension data were explained by variables in the multiple imputation model listed below. Monte Carlo Markov Chain imputation (Schafer, 1997) with predictive mean matching was performed with SPSS (v22) using variables that: (1) explained variance in the observed data for the variables with missingness, (2) were used in planned analyses, and (3) were used in potential control analyses to ensure the validity of pooled results from 10 multiply imputed datasets (Raghunathan et al., 2001). Pooling of results from the 10 imputed datasets was performed using the standard principle of combining point and variance estimates from imputed data (Little and Rubin, 2002). The point estimates are averages of the 10-point estimates from analysis of each imputed dataset, whereas the variance estimates account for both within and between imputation variability. Finally, the multiple imputation model included the four behavioral measures described above, dummy variables for site, age [and a quadratic age (age^2^) variable because of nonlinear age-related changes in regional gray matter volume], reading disability group, gender, a gender by reading disability interaction, and total gray matter volume.

#### Sample heterogeneity: discriminant function analysis

Retrospective multisite samples are likely to include groups of heterogeneous cases because of differences in sampling across sites and/or definitions of reading disability, as described in the Introduction. For each multiply imputed dataset, discriminant function analysis was performed using the Word Attack, Word Identification, and Passage Comprehension scores to identify cases that were not consistently classified as reading disability or control cases. The correctly classified cases were used for comparing the gray matter measures between the reading groups.

#### Control for OWL LD

Ideally, measures of expressive and receptive language would be used to control for OWL LD (or specific language impairment), but these data were not available for this retrospective dataset. We considered other defining features of OWL LD to identify and exclude these cases from the gray matter comparisons between reading disability groups. Specifically, we used measures of verbal comprehension and total gray matter volume to identify possible OWL LD cases based on evidence of significantly lower verbal comprehension (Snowling et al., 2000; Silliman and Berninger, 2011; Girbau-Massana et al., 2014) and significantly lower total gray matter volume in OWL LD cases compared with controls (Leonard et al., 2002; Girbau-Massana et al., 2014). The rationale for using these variables to identify potential cases of OWL LD is further supported by evidence that children with reading disability and lower receptive language performance are more likely to have significantly lower-left and right cerebral hemisphere volumes compared to children with reading disability with higher receptive language function (Kibby et al., 2009).

A brain size effect on verbal comprehension in children with OWL LD appears to be present early in life (Whitehouse et al., 2012), but we considered the possibility that some children can exhibit low brain volume and have relatively normal oral and written language skills. We also considered evidence that poor reading skills can lead to lower verbal comprehension in older children who must read to learn (Ramsden et al., 2013). Thus, children classified as OWL LD in the current study had to have low values for both verbal comprehension and total gray matter volume.

The classification of children with OWL LD was based on the mean group differences reported in the OWL LD literature. In particular, children with OWL LD exhibit verbal comprehension scores ∼1 SD below the mean of the population (∼16^th^ percentile; Snowling et al., 2000; Silliman and Berninger, 2011; Girbau-Massana et al., 2014) and brain volume measures ∼.67 SD below the mean of control cases (∼25^th^ percentile of the sample; Leonard et al., 2002; Girbau-Massana et al., 2014). Thus, cases below the 16^th^ percentile in verbal comprehension and below the 25^th^ percentile in total gray matter volume for the sample were classified as having possible OWL LD (*n* = 10) and excluded from group comparisons in control analyses. We used 25^th^, 30^th^, and 37^th^ percentile thresholds for both variables to determine the extent to which the ROI results were affected by excluding cases for these more liberal thresholds for defining OWL LD (*n* = 12, 15, and 21 respectively). Although this approach may not have identified every case with OWL LD, it helped to characterize the extent to which group differences in the gray matter data could be attributed to oral and written language problems when these cases were removed from the group comparisons.

#### ROI gray matter comparisons

The three ROI gray matter variables identified from the meta-analysis were first correlated with the behavioral data to determine the extent to which they exhibited significant associations across the multisite sample (*n* = 293). For example, we examined the extent to which the average gray matter volume in a left superior temporal sulcus region from the meta-analysis was significantly predictive of Passage Comprehension scores. Thus, we used a dimensional approach to examine the extent to which the broad range of reading scores related to the ROI measures across the entire dataset before and after accounting for reading group. As described above, the ROI gray matter volumes were also compared between reading groups to determine the extent to which ROI gray matter group differences from the meta-analysis results were also observed in the multisite data. These group comparisons did not assume homogeneity of variance as increased variance was observed in the reading disability group compared to control group. This analysis included only cases identified as control or reading disabled in the discriminant function analysis. This typical group difference design therefore included relatively more homogenous reading groups that were clearly distinct in their reading skills. Bonferroni correction was used to control for multiple comparisons for correlation and group difference analyses.

#### Voxel-based gray matter group differences

Voxel-based morphometry was performed to determine the extent to which there were reading disability group differences in gray matter volume across brain regions that were not represented by the meta-analysis ROI. The reading groups identified with the discriminant function analysis were used for this analysis.

The Levene test for homogeneity of variance was first used to examine the extent to which there were reading disability group differences in gray matter variance that could impact the likelihood of observing group differences in gray matter means between the reading groups. Levene tests were performed for each gray matter voxel within a 20% probability mask using R (library “car”). SD maps also are shown below to demonstrate regions of high variance before and after accounting for nuisance covariates. This analysis was designed to determine the extent to which increased anatomical variance occurred in brain regions that are inconsistently reported in gray matter studies of dyslexia and that might impact the likelihood of observing group differences.

Reading disability group differences in voxel-based gray matter volume were examined while controlling for age (and the age^2^ variable), gender, and research site. The age^2^ variable was included to account for nonlinear changes in gray matter that can occur during childhood (Wilke et al., 2007).

The default settings for group comparisons in SPM assume non-homogeneity of variance, and thus the group comparisons were appropriate for gray matter regions exhibiting group differences in gray matter variance. All analyses were limited to voxels with at least a 20% probability of gray matter across cases based on the DARTEL study-specific gray matter template. Voxel-based results from the 10 imputed datasets were pooled, as described in the multiple imputation section, to obtain group results. Statistical significance for these more exploratory analyses was defined with a familywise error-corrected, *p* < 0.05 peak voxel threshold. We did explore the possibility of gender effects using a gender by reading disability interaction term based on gender findings in dyslexia gray matter studies (Altarelli et al., 2013, 2014; Evans et al., 2014), but these results were not significant and are not described further.

## Results

### Meta-analysis of reading disability gray matter studies

**Figure 1.**
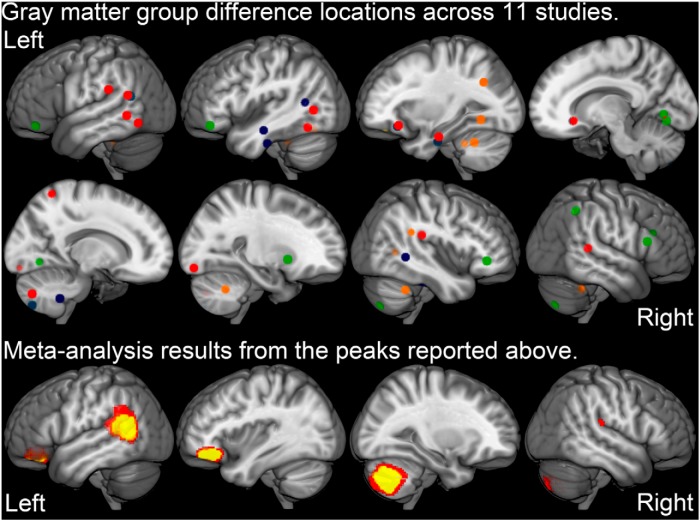
**Meta-analysis results**. Widespread reading disability group differences in gray matter volume reported across 11 voxel-based studies of reading disability are displayed on the MNI T1 image (top). SDM meta-analysis results demonstrated relatively consistent effects within left superior temporal sulcus, left orbitofrontal cortex, and right cerebellar hemisphere (bottom; red, *p* < 0.005, yellow *p* < 0.001, uncorrected). Jednoróg et al. (2015) was not included in the meta-analysis and did not observe cortical effects for *p* < 0.001 peak and *p* < 0.05 cluster extent thresholds. The yellow clusters were normalized into the study-specific DARTEL space of the multisite data to extract average gray matter volume estimates for the dimensional and group difference analyses.

Figure 1 presents the varied spatial locations of gray matter group differences across the 11 studies included in the meta-analysis. Significantly lower gray matter volume in reading disability compared with control cases was observed in left orbitofrontal cortex/pars orbitalis of the inferior frontal gyrus/(MNI: −38, 36, −14; *Z* = 2.49), left posterior superior temporal sulcus/middle temporal gyrus (MNI: −56, −56, 8; *Z* = 2.74), and right cerebellar hemisphere (MNI: 24, −68, −42; *Z* = 2.36) regions across studies. There were no regions exhibiting significantly greater gray matter volume in the reading disability compared with controls across studies. There were too few studies to obtain reliable associations between varied methods or sampling approaches across studies (eg, gender ratio) and the varied locations of reported effects.

### Associations between ROI gray matter and behavior

There was a wide range of reading skills among the reading disability and control cases, as demonstrated in Table 2, which motivated a dimensional data analysis approach. Table 4 shows that the left orbitofrontal, left posterior superior temporal sulcus, and right cerebellar hemisphere ROI exhibited significant associations with passage comprehension (*r* = 0.17 to *r* = 0.20) and survived Bonferroni correction for 12 comparisons (*p* = 0.004 to *p* = 0.0006) after controlling for gender, age, age^2^, and research site. These passage comprehension results were nearly identical when excluding cases with multiply-imputed passage comprehension data (eg, left superior temporal sulcus × passage comprehension: *r* = 0.20, *p* = 0.001). These associations were diminished when controlling for reading group (Passage Comprehension by left orbitofrontal cortex, partial *r* = 0.14, *p* = 0.019, by left superior temporal sulcus, partial *r* = 0.11, *p* = 0.054, and right cerebellar hemisphere, partial *r* = 0.13, *p* < 0.028). The inclusion of reading disability cases with relatively high Passage Comprehension scores for their group explained why these structure–function associations were not completely explained by reading group.

**Table 4.** Pearson correlations between gray matter regions from the meta-analysis and behavioral measures across all 293 cases

	Word attack	Word identification	Passage comprehension	Verbal comprehension
Left Orbitofrontal Cortex (resid)	0.17**	0.17**	0.20***	0.11
Confidence Intervals	0.05–0.29	0.05–0.28	0.07–0.32	0.01–0.21
Left Superior Temporal Sulcus (resid)	0.16*	0.17**	0.19**	0.10
Confidence Intervals	0.03–0.27	0.06–0.28	0.08–0.30	0.00–0.21
Right Cerebellar Hemisphere (resid)	0.15*	0.11	0.17**	0.16**
Confidence Intervals	0.02–0.27	−0.01 to 0.24	0.04–0.29	0.05–0.27
Total Gray Matter (resid)	0.20***	0.19***	0.23***	0.21***
Confidence Intervals	0.07–0.32	0.06–0.31	0.11–0.34	0.10–0.32

Shaded cells indicate correlations that survive Bonferroni correction for the number of ROI and behavior correlations (0.05/12, *p* < 0.004); (resid) gray matter volume residualized for site, gender, age, and age^2^.

*p < 0.05, **p < 0.01, ***p < 0.001.

### Reading group ROI gray matter differences

Again, discriminant function analysis was used to establish behaviorally distinct reading groups using the Passage Comprehension, Word Identification, Word Attack, and Verbal Comprehension scores for the group comparisons. An average of 7.06% reading disability cases (*n* = 11.6) were incorrectly classified as controls. An average of 17.36% control cases (*n* = 22.4) were incorrectly classified as reading disabled across the 10 imputed datasets. This analysis yielded 150 reading disability and 105 control cases for the gray matter comparisons that exhibited significant group differences (all *p* < 0.001) in Word Attack, Word Identification, Passage Comprehension, and Verbal Comprehension (Table 2).

**Figure 2.**
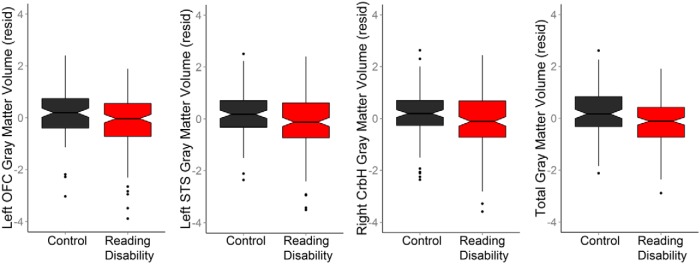
**Reading disability group differences in gray matter within meta-analysis ROI (*n* = 255)**. Significantly lower gray matter volume [adjusted for age, age^2^, gender, and site (resid)] was observed in reading disability cases compared to controls within left OFC (including pars orbitalis), and left STS. There were not significant reading disability group differences in right cerebellar hemisphere (CrbH) ROI gray matter volume, perhaps because of the increased gray matter volume variance in the reading disability group. The ROI group differences in gray matter volume were statistically dependent on total gray matter volume.

The control group exhibited significantly more gray matter volume than the reading disability group for the left orbitofrontal (*t*_(246.9)_ = 2.70, *p* = 0.007; Cohen’s *d* = 0.34) and left superior temporal sulcus (*t*_(248.8)_ = 2.86, *p* = 0.005; Cohen’s *d* = 0.37) ROI after Bonferroni correction for three comparisons (*p* < 0.017; Fig. 2), but not for the right cerebellar hemisphere ROI (*t*_(242.2)_ = 1.79, *p* = 0.075; Cohen’s *d* = 0.24). These comparisons were performed without assuming equal variances because there was increased variance in the reading disability group compared with the control group for these three variables (Levene test: *p* = 0.071, *p* = 0.017, *p* = 0.063, for each region, respectively).

We then examined the extent to which children with evidence of OWL LD accounted for the reading disability group differences in orbitofrontal cortex (OFC) and superior temporal sulcus (STS) gray matter volume. Table 5 shows a small change in Cohen’s *d* effect size for the ROI group differences with the exclusion of potential OWL LD cases. Moreover, there was relatively limited impact of more liberal classification criteria for OWL LD despite the decreasing sample size (Table 5). These results show that the OFC and STS group differences required a large sample size given the small effect sizes. These results also suggest that OWL LD cases can contribute to group differences for these gray matter measures rather than obscure or drive the group differences.

**Table 5.** Cohen’s *d* effect sizes for the reading disability group comparisons with and without cases exhibiting evidence of OWL LD based on combined verbal comprehension and total gray matter volume values that were below the combined percentile cutoffs

	Left OFC	Left STS	Right CrbH
No cases removed (*N*=255)	0.34	0.37	0.24
Cases <15^th^/25^th^ percentile removed (*N*=245)	0.31	0.33	0.18
Cases <25^th^ percentile removed (*N*=243)	0.28	0.29	0.16
Cases <30^th^ percentile removed (*N*=240)	0.28	0.26	0.15
Cases <37^th^ percentile removed (*N*=234)	0.28	0.27	0.12

The 15^th^/25^th^ percentile thresholds for classification of OWL LD were used for verbal comprehension and total gray matter volume, respectively. These thresholds were chosen based on the size of group differences reported in the literature for those variables. Otherwise, the same percentile threshold was used for both variables. The Cohen’s *d* values were calculated based on the mean and SD of the ROI gray matter volumes for each reading disability group.

Finally and most importantly, an ANCOVA demonstrated that there were no significant reading disability group differences in OFC and STS gray matter volume when controlling for total gray matter volume (OFC group: *F*_(1,254)_ = 0.83, n.s.; STS group: *F*_(1,254)_ = 0.16, n.s.). This result was consistent with the significant difference in total gray matter volume between reading groups (*F*_(1,254)_ = 10.41, *p* = 0.001; Cohen’s *d* = 0.43). Thus, the reading group differences in OFC and STS gray matter volume were because of cases with low total gray matter volumes irrespective of cases that also had low verbal comprehension.

### Voxel-based gray matter comparisons

**Figure 3.**
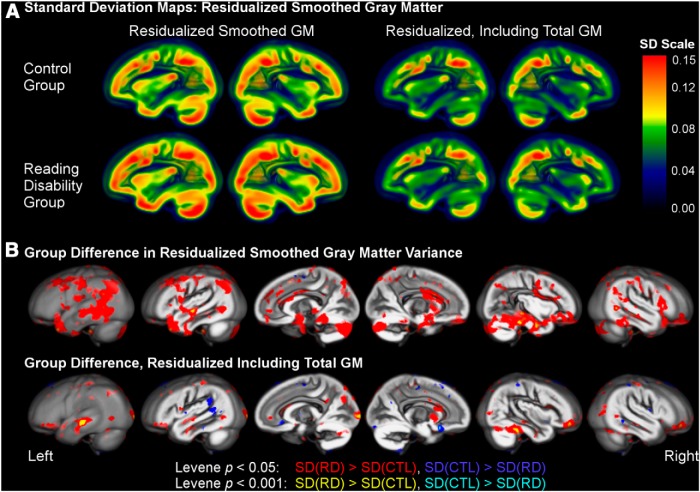
**Gray matter variance in control and reading disability groups. *A***, Representative sections shows standard deviations across voxels for the smoothed gray matter images (residualized smoothed GM: after removing variance for gender, age, and research site; corrected for total GM: after removing variance for gender, age, research site and total gray matter volume). ***B***, A voxelwise Levene test (uncorrected for multiple comparisions) demonstrated brain regions where there were reading disability group differences in gray matter variance [red: reading disability (RD) > control (CTL); blue: CTL>RD].

Voxelwise Levene tests of the homogeneity of variance indicated significantly greater variance in the reading disability group compared to the control group, before and after accounting for total gray matter volume. This increased variance in the reading disability group includes superior temporal, supramarginal, inferotemporal, occipital, and cerebellar regions that have been implicated in dyslexia. Figure 3 shows these variance effects and demonstrates relatively more pronounced gray matter variance in the reading disability group compared to the control group. This increased variance is important when considering the magnitude of the group differences in voxel-based gray matter described below.

**Figure 4.**
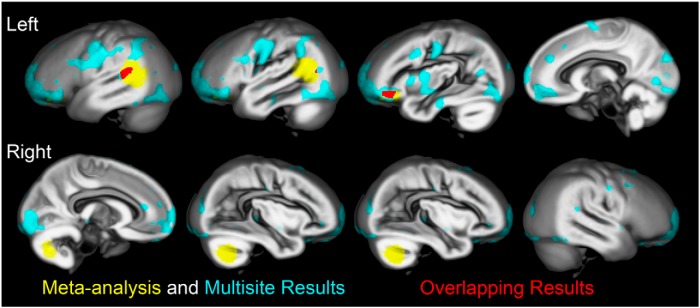
**Multisite and meta-analysis results**. The multisite voxel-based reading group comparison results (cyan) are presented with the meta-analysis results (yellow) from voxel-based dyslexia studies on the study specific DARTEL gray matter template (all clusters *p* < 0.001, uncorrected). The multisite group comparison included covariates for age, age^2^, gender, and research site. The left hemisphere overlap of results (red) from each analysis falls within the left posterior superior temporal sulcus and left orbitofrontal cortex. All cyan clusters were no longer present using the *p* < 0.001 uncorrected threshold when total gray matter volume was included as a covariate.

Exploratory voxel-based comparisons demonstrated widespread lower gray matter volume estimates in reading disabled compared with control cases for an uncorrected *p* < 0.001 threshold when controlling for gender, age, age^2^, and research site. Figure 4 shows that these results overlap the space of the left orbitofrontal and superior temporal sulcus meta-analysis ROI. There were no results that survived familywise error correction despite the relatively large sample size. In addition, removing cases with evidence of OWL LD (<16^th^ percentile verbal comprehension and <25^th^ percentile total gray matter volume) did not uncover any new results that had been obscured by the OWL LD cases. Finally, there were no voxel-based group differences at the *p* < 0.001 uncorrected threshold when the total gray matter volume variable was included in the model. These results are consistent with results in Tables 4 and 5, which demonstrate a strong influence of total gray matter volume on the multisite results.

## Discussion

Reading disability and poor cloze reading comprehension occurs with low gray matter volume in left orbitofrontal gyrus/pars orbitalis and left posterior superior temporal sulcus/middle temporal gyrus regions compared with typical readers based on a meta-analysis of the extant voxel-based reading disability literature and direct analysis of multisite data. These effects were present despite reading disability group differences in gray matter variance. These effects also were present when controlling for research site, age, and gender, but do require large sample sizes and children with reading disability who have relatively low total gray matter volume. These total gray matter volume-dependent results appear to reflect a primary source of reading difficulty that rises above other sources of atypical development among children with a complex disorder that can have multiple etiologies.

### Varied behavioral profiles and sampling

Reading disability samples are often collected with varied sampling approaches that can yield samples with quite different behavioral profiles and therefore impact study results. For example, Eicher et al. (2014) observed relatively consistent genetic effects of DYX2 (KIAA0319) alleles on reading, language, and IQ measures, but the strength and pattern of associations varied across four samples. This sampling and behavioral heterogeneity issue could explain why so many different structural findings have been reported with limited replication (Fig. 1). The research site differences in real word reading among children with reading disability in the current study further supports the premise that children with quite different reading profiles are included in neuroimaging studies of dyslexia.

The current study included a discriminant function analysis of the behavioral data to select cases for control and reading disability groups that were clearly different in their reading skill abilities (Table 2). Despite these attempts to control for heterogeneity, the reading disability group was still composed of cases with quite varied behavioral (Table 2) and anatomical (Fig. 3) profiles. Nonetheless, reading disability group differences in left orbitofrontal and left posterior superior temporal sulcus gray matter volume were observed. These findings appear to be relatively robust compared with findings implicating other brain regions in reading disability. Moreover, the results are consistent with genetic evidence that KIAA0319 alleles confer risk for impaired reading and language functions across different samples (Eicher et al., 2014).

### Potential genetic explanations for the gray matter findings

#### Left orbitofrontal/pars opercularis finding

The KIAA0319 minor risk allele for SNP rs9461045 has been associated with cortical thickness in left orbitofrontal cortex across a normative sample of 322 subjects that ranged from 3 to 22 years of age (Eicher et al., 2015). This result is consistent with the meta-analysis and direct data analysis findings from the current study that people with reading disability have lower left orbitofrontal gray matter volume. In addition, KIAA0319 SNP polymorphisms (rs2038136 and rs2038137) have been linked to the functional coherence of an orbitofrontal/pars orbitalis/inferior frontal sulcus and parietal network (Jamadar et al., 2013). Together, these results suggest a direct link between atypical development of orbitofrontal/pars orbitalis function and structure that limits reading development.

There is a possibility that despite these genetic associations, the orbitofrontal findings are indirectly related to reading disability. The findings described here could be explained, at least in part, by comorbid attention deficit (Sexton et al., 2012) and/or the social emotional consequences of having a reading disability (Martínez and Semrud-Clikeman, 2004; Snowling et al., 2007) on orbitofrontal cortex (van't Ent et al., 2007; Fernández-Jaén et al., 2014). In addition, externalizing behaviors impact the likelihood that teachers refer children with learning disabilities for special education services (Lloyd et al., 1991) and externalizing behaviors occur with relatively lower cortical thickness in left orbitofrontal cortex (Ameis et al., 2014). Early childhood studies may help to differentiate these potential direct and/or indirect effects on orbitofrontal development.

#### Left posterior superior temporal sulcus finding

The gray matter reading disability group differences in the left posterior superior temporal sulcus are consistent with the premise that reading disability occurs in children with atypical development of posterior temporal regions (Gabrieli, 2009) that support phonologic processing (Price, 2012). Moreover, the posterior superior temporal sulcus is a target for arcuate fasciculus projections where a DCDC2 allele (rs793842), which confers risk for dyslexia (Meng et al., 2005), occurs with lower temporoparietal white matter volume within a fiber distribution that appears to terminate in the posterior superior temporal sulcus (Darki et al., 2012, their Fig. 3C). Atypical white matter and the DCDC2 risk allele have also been observed in cases with low left middle temporal gyrus cortical thickness (rs793842; Darki et al., 2014). Together, these findings suggest that DCDC2 risk alleles contributed to the superior temporal sulcus findings in the current study.

#### Additive multigenic variation

The left posterior superior temporal sulcus and orbitofrontal effects both appeared to be dependent on total brain volume in the current study. This observation is consistent with the location of ectopic neurons in orbitofrontal and superior temporal regions, as well as an apparent negative association between low brain weight and the frequency of ectopias in the small sample of postmortem brains from reading disabled males, who were studied by Galaburda et al. (1985). An additional spatial correspondence with the current results, as well as and the central sulcus region in Figure 4, is that these regions show pronounced rates of growth in gestation during a critical period of cortical folding (20–28 weeks; Rajagopalan et al., 2011) when the maximal number of cortical neurons is reached (de Courten-Myers et al., 1996). Thus, it is reasonable to hypothesize that genes associated with dyslexia and migrational error in animal models, such as KIAA0319 (Platt et al., 2013) and DCDC2 (Meng et al., 2005), contribute to orbitofrontal and superior temporal sulcus findings in people with reading disability.

The orbitofrontal/pars orbitalis and superior temporal sulcus results also are aligned with the diffusion imaging literature implicating arcuate fibers of the superior longitudinal fasciculus in dyslexia (Boets et al., 2013). Migrational errors in regions that are connected by the superior longitudinal fasciculus would be expected to produce atypical diffusional properties because fibers have lost their targets and/or the patterns of projection are less organized. In support of this premise, a KIAA0319 risk allele (rs6935076) has been associated with lower temporoparietal white matter volume (Darki et al., 2014). Moreover, spatial variance in the location of migrational errors that leads to atypical structure in a large tract could explain why diffusion-imaging findings from dyslexia studies have been relatively more consistent than gray matter findings.

The KIAA0319 and DCDC2 findings described above suggest that together these genes impact the development of orbitofrontal and superior temporal regions and may have additive effects. Interestingly, reading skills appear to be particularly impaired when KIAA0319 and DCDC2 risk haplotypes are both observed (Powers et al., 2013). This observation is generally consistent with the observation from the current study that cases with the poorest Passage Comprehension scores had relatively low gray matter volumes. Given the relatively low frequency of people having both KIAA0319 and DCDC2 risk haplotypes (see www.deeveybee.blogspot.org, June 2013), a small number of cases with pronounced atypical development would likely contribute to reading group gray matter differences. This observation is consistent with the increased variance in the reading disability sample that appeared to be dependent, in part, on low total gray matter volume in a subset of reading disability cases. Moreover, this would suggest that widespread and pronounced migrational errors are necessary to observe gross morphological effects that are spatially specific using normalized space voxel-based approaches and that rise above a statistical threshold in anatomically heterogeneous samples.

### Inconsistencies in the meta-analysis and direct analysis results

The left superior temporal sulcus and left orbitofrontal cortex results were spatially overlapping across the meta-analysis and direct data analysis results. While there was a trend for group differences in right cerebellar hemisphere gray matter volume, the direct data analyses did not strongly replicate cerebellar findings from dyslexia studies that have been reported with relative consistency (Pernet et al., 2009; Stoodley, 2014) and that are demonstrated by the meta-analysis results (Fig. 1). One explanation for these results is the increased gray matter variance across cerebellar regions in the reading disability compared with the control group (Figs. 2, 3). This variance result replicates an observation that cerebellar gray matter measures are more extreme in dyslexic compared with control cases (Pernet et al., 2009) and suggests that anatomical variance impacts the likelihood of observing cerebellar effects in reading disability studies.

Increased gray matter variance in the reading disability group compared to control group was observed not only in the cerebellum but also across the brain. Left and right inferotemporal regions exhibited increased variance in the reading disability group, even after controlling for the impact of total gray matter on these group differences (Fig. 3). This is an important observation because reports of inferotemporal gray matter differences (Linkersdörfer et al., 2012) could be inconsistent because of increased anatomical variance in reading disability samples. We did observe reading disability group differences in inferotemporal cortex (*p* < 0.001, uncorrected) before controlling for total gray matter volume, which suggests that inferotemporal group differences are at least partially dependent on the inclusion of cases with low total gray matter volume.

The variance results suggest the possibility that anatomical variance is a defining feature of reading disability because of dysregulated cortical patterning or varied location of migrational errors. Perhaps more simply, the variance findings may reflect the etiological heterogeneity of reading disability. For example, Männel et al. (2015) have reported that children with dyslexia who have a dyslexia risk allele in the TNFRSF1B gene also have lower verbal working memory and higher left posterior STS gray matter volume. The risk allele(s) that some children with dyslexia carry therefore appears to differentially impact the amount of STS gray matter volume and would lead to increased anatomical variance.

Differential genetic effects on gray matter volume (eg, DCDC2 vs TNFRSF1B) would reduce replication across dyslexia studies, perhaps as demonstrated by the varied spatial locations of reported effects in Figure 1. This possibility is important when considering results from the meta-analysis localizer approach that was used in this study. For example, Linkersdörfer et al. (2012) reported meta-analysis results that dyslexia cases were more likely to have lower right SMG gray matter compared with controls. We also observed this effect when studies from two of the contributing sites for this project were included in the meta-analysis. This right SMG result (and an inferotemporal result discussed above) were no longer present when excluding these two studies to ensure that the direct data analysis results were independent of the meta-analysis results. Although the left STS, left OFC, and right cerebellar effects were insensitive to the inclusion or exclusion of these two studies, it is possible that there are other brain regions that are important for understanding reading disability and that were not included in our meta-analysis results. This idea might be supported by the widespread voxel-based results shown in Figure 4, but all of these effects (*p* < 0.001, uncorrected) were diminished after controlling for total gray matter volume.

There were some regions where controls exhibited increased gray matter variance compared to the reading disability cases (eg, left posterior end of the Sylvian fissure), but the vast majority of Levene test differences were driven by increased variance in the reading disability group. This observation is consistent with observations from Leonard et al. (2006) and Pernet et al. (2009) suggesting that we should focus on the anatomical variance in reading disability samples when considering etiology for a complex disorder and when thinking about identifying children who are at risk for reading disability. This variance perspective could be particularly important for studying cases with dyslexia who have migrational errors that may not always fall in the same voxels across brain images (see the varied spatial position of ectopias and dysplasias from Galaburda et al., 1985, their Figs. 1, 4, 5, 6).

## Conclusions

There is consistent, but modestly lower gray matter volume in left orbitofrontal cortex/pars orbitalis and left posterior superior temporal sulcus/middle temporal gyrus regions in people with reading disability based on meta-analysis and direct data analysis of multisite data. These findings are further supported by evidence of reduced cortical thickness across these same regions in children with dyslexia (Clark et al., 2014), but would not have been reported in this study given the uncorrected *p* < 0.001 effects for the direct data multisite analysis or if total gray matter volume was controlled (Jednoróg et al., 2015) without the meta-analysis results to support these effects. The local gray matter volume findings reported here appear to be dependent on total gray matter volume effects that increased the anatomical variance among children with reading disability. These total brain volume and variance results are perhaps observed because of widespread migrational errors that are concentrated in perisylvian regions (Galaburda et al., 1985) and/or because of atypical arcuate fasciculus development (Boets et al., 2013; Wandell and Yeatman, 2013). Together, the results suggest that the brain volume effects associated with the left superior temporal sulcus and orbitofrontal gyrus development have the potential to isolate primary etiologies for reading disability that we predict include effects related to KIAA0319 and DCDC2 risk alleles.

## Synthesis

The decision was a result of the Reviewing Editor Alfonso Represa and the peer reviewers coming together and discussing their recommendations until a consensus was reached. A fact-based synthesis statement explaining their decision and outlining what is needed to prepare a revision is listed below. The following reviewers agreed to reveal their identity: Gerard Ridgway

This voxel-based morphometry study of reading disability obtains a large sample size of 164 cases and 129 controls by combining data from 6 sites. It complements the whole-brain voxel-wise analysis with regions of interest defined from a coordinate-based meta-analysis of 11 previous studies, comprising 218 control and 244 dyslexia cases. The main regions implicated are left posterior superior temporal sulcus / middle temporal gyrus and left orbitofrontal gyrus / pars orbitalis. The manuscript also demonstrates increased anatomical variance in the reading disability group over much of the brain. The study shows that even the significant effects (using the meta-analysis regions of interest) are slight (Fig.2) consistent with the only other similarly large study (Jednoróg et al., 2015), though the present manuscript is not as pessimistic about the previously reported small-sample results as that paper. The methods seem sound, and the manuscript is generally well written, although I think the use of multiple imputation and a discriminant function subgroup add substantial complexity without seeming to have an important impact on the results. I think some aspects should be more carefully discussed.

Major points

1. Relation of global and local volumetric differences.

It is noted in the abstract (and a similar statement on page 19) that "Gray matter volume in [localised] regions explained reading group differences in total gray matter volume". This interpretation is opposite to the usual convention in brain-imaging, where one typically asks whether the local differences persist after adjusting for the total/global measures (i.e. allowing the global to explain the local, rather than vice versa). It would be helpful to present results adjusted for total GM (at least in supplementary material, if not the main figures and tables).

Whether one interprets the primary effect as local, with consequent contribution to total GM, or as a primarily global effect which is widely distributed and just happens to reach significance in certain local areas, is a difficult question, perhaps requiring structural equation modelling or other advanced techniques to address properly. In Table 3, total GM has the strongest correlations with each of the behavioral measures, which perhaps argues against the interpretation of the local effects as the primary cause of the associations. The statement (p20) "gray matter volume in the left orbitofrontal and left superior temporal sulcus was the reason for the differences in total gray matter volume or are at least sensitive markers of total gray matter volume" seems too bold, given that total GM had the strongest correlations in Table 3. If nothing else, I think this issue warrants further discussion.

2. Partial or semi-partial correlations.

It is unclear ("after controlling ...", page 11), whether adjustment for age etc. is performed by adjusting the volumetric data and associating with the unadjusted behavioral measures, or whether both are adjusted. The use of "res" in Table 3 for volumetric measures (but not behavioral) suggests that a form of semi-partial correlation is used, instead of the partial correlation that would result when both are adjusted. Since age (etc.) might well explain some behavioral variance, I would prefer partial to semi-partial correlations. This is discussed briefly in the following:

http://www.ncbi.nlm.nih.gov/pubmed/25843619

3. Control for potential SLI / OWL LD effects.

The methods section is rather unclear about this aspect. In the results (p20), it is referred to as "controlling for the shared total gray matter and Verbal Comprehension variance", but this is unclear. What actual regressor(s) is/are used to control for this "shared variance"? Is it e.g. the result of predicting one/each variable using the other? The claim in the discussion that the authors "also controlled for evidence of SLI / OWL LD without removing variance in Verbal Comprehension that could be due to poor reading" seems too bold; can the shared variance regressor(s) really be considered as a valid proxy of SLI / OWL LD? Can it really avoid removing variance related to poor reading?

4. Differences from prior meta-analyses.

A previous meta-analysis (Richlan et al., 2013) included 9 studies, 8 of which are shared in the present meta-analysis (Brown et al., 2001; Brambati et al., 2004; Eckert et al., 2005; Silani et al., 2005; Vinckenbosch et al., 2005; Kronbichler et al., 2008; Menghini et al., 2008; Steinbrink et al., 2008). One study from the previous meta-analysis has been excluded here (Hoeft et al. 2007 - http://www.ncbi.nlm.nih.gov/pubmed/17360506), and it would be helpful if the authors could indicate why. One study (Raschle et al., 2011) included in the present meta-analysis was considered but excluded from the previous meta-analysis, "because they studied prereading kindergarteners with a family history of developmental dyslexia rather than diagnosed dyslexics", a second study (Black et al., 2012 - http://www.ncbi.nlm.nih.gov/pubmed/22023744) was excluded for the same reason. Lastly two studies published since the previous meta-analysis have been included here (Jednorog et al., 2013; Tamboer et al., 2015). It would be helpful if the authors could discuss the inclusion-related differences in more detail, e.g. what peaks were reported in the studies that were present in only one of the two meta-analyses, and how do these relate to the results of the meta-analyses?

More importantly, Linkersdörfer et al. (2012) is only cited once in the manuscript (page 4) without much discussion, despite its obvious relevance. It includes 9 studies, 7 of which are included in the present meta-analysis (Brown et al., 2001; Brambati et al., 2004; Eckert et al., 2005; Vinckenbosch et al., 2005; Kronbichler et al., 2008; Steinbrink et al., 2008; Raschle et al., 2011). Two studies included in Linkersdörfer et al. (2012) but not in the present submission are Hoeft et al. (2007) and Pernet et al. (2009). The studies included here but not in Linkersdörfer et al. (2012) are Jednorog et al. (2013) and Tamboer et al. (2015). Despite having 7 studies in common with the present meta-analysis, the results seem to be quite different (e.g. left and right supramarginal gyrus, left fusiform gyrus, among other regions). I think this requires discussion. It is noted on page 7 that the present "methods are consistent with those used by Richlan et al. (2013)", which raises the question of how sensitive the results are to the details of the meta-analysis; would results of the present meta-analysis be more similar to Linkersdörfer et al. (2012) if methods consistent with that study were employed in the present meta-analysis?

5. Clarity regarding independence.

On p7 it is stated that "Excluding these 2 studies from the meta-analysis ensured that the meta- and direct data analyses described below were performed with completely independent datasets." However, the footer of Table 1 has the note "Sites 2 and 5 included cases whose data contributed to reported effects represented in the meta-analysis. The meta-analysis results were not substantively different when excluding the effects from these sites." This note suggests that although the meta-analysis was performed with and without these sites, the main reported results included them. Later though, on p17, it is implied ("also performed with") that the main results exclude these studies. Please clarify.

Minor points

- Abstract (and several subsequent occasions), I think the term "reading group differences" should be replaced with either just "group differences" or "reading disability group differences".

- pages 3 and 4, references to "the population" are slightly ambiguous; does this refer to the e.g. USA, all English-speaking countries, or the whole world?

- p5, the range of sample sizes quoted "(N = 8-46, median = 13)" seems to exclude the largest study (Jednorog et al., 2015). It is not clear why this study is excluded from the meta-analysis.

- p5, the mention of "statistical thresholds p &lt; 0.05 to p &lt; 0.0001 uncorrected for multiple comparisons" seems to be contradicted by the tables in Linkersdörfer et al. (2012) and Richlan et al. (2013), which both indicate that some of the studies used correction for multiple comparisons (either based on peak-height or cluster-extent). I apologise for not taking time to check the original studies myself.

- p5 (etc.), I find the abbreviation "SLI / OWL LD" rather unwieldy; is it a standard one, or could the authors define their own shorter abbreviation for this?

- p6, it is unclear exactly what is meant by the term "confirmatory power" (the wording implies it is distinct from statistical power).

- Table 1, it would be helpful to include the "(n=Control/RD)" numbers for the "All Cases" and "Selected Cases" rows.

- p10, it is unclear why it would be important to have "common header information (e.g., bounding box size)" and/or whether this relates to voxel-size (which is more than just header information, since it would require resampling the image data to obtain common dimensions).

- p10, it is somewhat surprising to see that the "SPM8 New Segment algorithm" was used after the mention of the VBM8 toolbox (which uses its own different segmentation algorithm). Were both tried? Were they consistent? Could other results be included in Supplementary Material?

- p10, smoothing will help to make data more normally distributed, but will not "ensure" normality as claimed.

- p11, details are needed for the method of "normalizing the SPM a priori gray matter image to the DARTEL gray matter template", e.g. is this affine or nonlinear?

- p12, the statement "because predictors of missingness could be identified, thereby meeting the assumption that the data were missing at random" seems somewhat counter-intuitive (predictable = random?) and might benefit from further explanation.

- p13, the discriminant function subgroup might be considered a form of circular inference; if the authors feel it is necessary, perhaps it could be complemented by providing full data-set results in supplementary material, corresponding to all subgroup results.

- p14, after the discriminant function subgroups have been introduced, the authors say "Thus, we also used a typical group difference design using relatively more homogenous reading groups that were clearly distinct in their reading skills"; this sounds confusingly like an addtional (third) group comparison?

- p17, three sets of results for passage comprehension are mentioned (original, excluding imputed cases, adjusting for group); I think it would be very helpful to present a scatter plot, where the points could indicate both group membership (e.g. circles and squares) and imputation status (hollow or filled), along with lines of best fit for each group.

- p17, the phrase "diminished, but relatively preserved" sounds odd (relative to what?). Perhaps reword as "diminished but persistent"?

- Figure 2's caption should clarify whether it shows results for the full data or discriminant function subgroups; perhaps add a second row of plots, so that both can be shown.

- p19, a measure of effect size (e.g. Cohen's d) should be given for the group difference results, rather than only t and p-values.

- p20, the statement that "This variance effect size was exaggerated" seems to hold only for the statistical significance, not the effect size (confidence interval), so probably reflects a general reduction in unexplained variance, rather than an increase in the variance inequality.

- p26, the section heading "additive genetic effects" doesn't seem quite right.

- p28, it is not clear what is meant by "modulating the gray matter volume values with relatively gross displacement fields" (perhaps "spatially normalising" rather than "modulating"?)

- p29, the statement that "regions of high variance could be regions where this variance would make it difficult to observe consistent reading group differences" is almost a tautology, since higher variance will inevitably reduce power. However, this is probably worth pointing out (though ideally reworded), since many wrongly consider VBM to be spatially "unbiased" (cf. ROI studies), despite warnings in the literature that power will vary over the brain.

- p36, there is an error in the citation for Price (2012).
